# Why Study the History of Neuroscience?

**DOI:** 10.3389/fnbeh.2019.00082

**Published:** 2019-05-22

**Authors:** Richard E. Brown

**Affiliations:** Department of Psychology and Neuroscience, Dalhousie University, Halifax, NS, Canada

**Keywords:** history, research, archives, libraries, museums

## Abstract

The history of neuroscience is the memory of the discipline and this memory depends on the study of the present traces of the past; the things left behind: artifacts, equipment, written documents, data books, photographs, memoirs, etc. History, in all of its definitions, is an integral part of neuroscience and I have used examples from the literature and my personal experience to illustrate the importance of the different aspects of history in neuroscience. Each time we talk about the brain, do an experiment, or write a research article, we are involved in history. Each published experiment becomes a historical document; it relies on past research (the “Introduction” section), procedures developed in the past (“Methods” section) and as soon as new data are published, they become history and become embedded into the history of the discipline (“Discussion” section). In order to be transparent and able to be replicated, each experiment requires its own historical archive. Studying history means researching books, documents and objects in libraries, archives, and museums. It means looking at data books, letters and memos, talking to scientists, and reading biographies and autobiographies. History can be made relevant by integrating historical documents into classes and by using historical websites. Finally, conducting historical research can be interesting, entertaining, and can lead to travel to out-of-the-way and exotic places and meeting interesting people.

## Introduction

I know of very few neuroscience programs that focus on the history of neuroscience. However, all research involves the study of history, even if it is only the history of a single research topic. For example, a research project on “The genetic basis of Long-Term Potentiation (LTP)” would involve some history of the discovery and relevant research on LTP. Likewise, a study on “The role of oxytocin in maternal behavior of *Peromyscus*” would involve a history of studies of the effects of oxytocin on behavior. In other cases, researchers testing a particular theory such as “The amyloid beta theory of Alzheimer’s disease” must discuss the history of this theory before they can make a new contribution to it. Researchers who use a particular research method, such as “single cell recording from the hippocampus” must know the history of the technique if they are going to improve upon it. Thus, all researchers are involved in studying the history of their particular research project and will summarize this history in the “Introduction” and “Discussion” sections of their research articles. Making space in a neuroscience program for a course on the history of neuroscience can be a hard sell, but neuroscientists with a strong historical understanding are more equipped to understand the context of their own work and its impact than those with no historical background. As noted by Shepherd (2010, p. 4):

“A historical perspective provides an education in how scientists are able to push past the limits of current concepts in order to fashion a new and more comprehensive understanding of the laws of nature.”

In 2008, Zoltán Molnár and I rediscovered a box of Charles Sherrington’s histological slides at Oxford University that had been neglected for over 70 years (Molnár and Brown, 2010). Sherrington is one of the most influential neurophysiologists in the history of neuroscience, winning the 1932 Nobel Prize in Physiology or Medicine (Eccles and Gibson, 1979). Considering this, it is incredible that these important historical scientific artifacts were unknown and inaccessible for such a long period of time. For every Sherrington’s box that is rediscovered and made accessible to scientific researchers and the public, many more historical objects are lost.

## What Is History?

“Everything but tomorrow is history”

*Grainger (1956; p. 81)*.

The definition of history and the methods used to conduct historical research are fraught with criticism and debate. Elton (1967) argued that historical research is the search for the objective truth about the past. Carr (1961), on the other hand, argued that history is a product of its own time; it is the interpretation of events and facts from the past through the specific lens of the historian’s own ideas and ideologies. Later, Samuel (1992) argued that history is *not* a record of the past, more or less faithful to the facts, but an invention or fiction of historians themselves. Edelman (2006) discusses the concept of scientific history from the point of view of scientists and historians. Indeed, there are so many aspects of the history of science that in some areas, such as physics, one can write a history of the history of science (Južnič, 2016). Thus, the term “history” has multiple meanings. History is the study of the past and includes the study of past events, the events connected with a particular person or discovery, a chronological record of an experiment, a historical document, or events that are no longer relevant to the present. History is a discipline, a method, and a personal memoir. Within neuroscience, all of these definitions apply and this article will discuss the relevance of different aspects of history to neuroscientists today.

### History as Memory

An approach to history that might appeal to neuroscientists is to treat history as memory, with all of its faults and flaws; its errors and omissions (Wachtel, 1986; Cubitt, 2007). The conception of history as a collective memory is a useful perspective for the study of neuroscience. Collective memory is a “form of memory associated with social groups, e.g., nations, families, etc”; it is a socially constructed history of the past (Poole, 2008). The recollections from one’s memory are fallible; a memory is constructed from a series of incidents connected in time and reconstructed from many components. It is not imprinted like a photograph. Memories are fragmented and very seldom do two people remember an event in the same way. Forgetting and false memories occur; emotions and opinions can distort memories and a recalled memory may become reconsolidated with erroneous information (Lee et al., 2017). Therefore, a “perfect” memory may include errors, omissions, distortions and false recollections (Loftus and Palmer, 1974; Schacter, 1996; Loftus, 2003). For example, in Dawkins’s (2013) memoir, *An Appetite for Wonder*, there is a group photograph that is captioned “The Animal Behavior Research Group after the move from Bevington Road.” That move occurred in 1971, but I am in that photograph, and I was only in the ABRG in 1976 and 1977. So, when was that photo taken? What was the event? My own memory of the event is vague. A third person featured in the photograph, Marian Dawkins, recalled that it was taken at a group picnic in November 1977. Clearly, even our own memories of events that we were involved in are subject to personal and unique distortions.

In considering the perspective of history as memory, the definition of history may shift towards the subjective perspective of Carr (1961) or Samuel (1992), and away from the objective perspective of Elton (1967). Overall, a memory can be seen as the raw material of history; whether it is a written or an oral recollection, it is the source from which historians draw information. History is thus a collection of individual and collective memories (Jimerson, 2003), which are the facts as we remember them. When the holders of these memories are gone, history becomes a cultural memory, which is the interpretation of these facts by someone who was not present at the event. Like any memory, history consists of a number of fragments linked together in time, with inevitable errors, confabulations, omissions, and errors of reconsolidation. Despite this, it is all we have. The definition of history as a collective memory can help to clarify the purpose of its study, as summarized by McNeill (1985):

“Historical knowledge is no more and no less than carefully and critically constructed collective memory. As such it can both make us wiser in our public choices and more richly human in our private lives.”

## History Is the Study of What Remains

**Figure 1:**
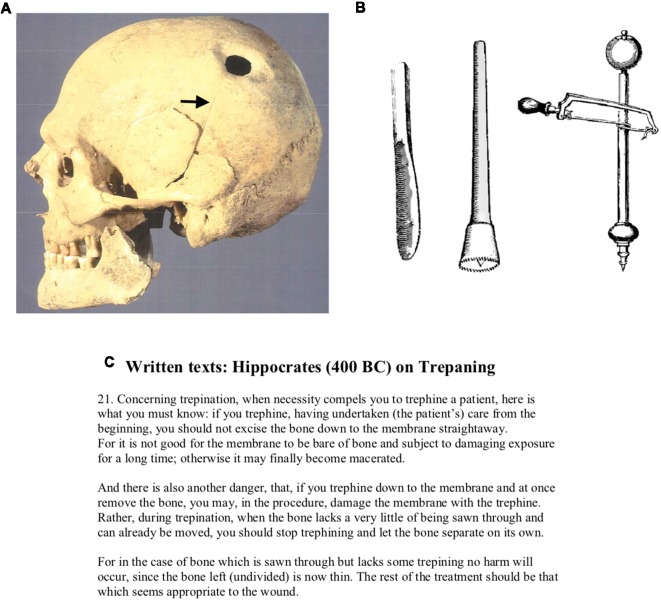
The three elements that are necessary to understand the significance of historical objects. In this case, the trephined skull **(A)** is the material object; the tools used for trephining **(B)** show how the holes might have been made, and the text from Hippocrates **(C)** gives a textual description of the instructions for using the tools to trephine the skull. **(A)** The trepanned skull of Chios. The arrow points to the healed linear fracture associated with the bur hole. Used with permission of the copyright holder: Copyright Hellenic Ministry of Culture and Sports, Ephorate Antiquities of Chios, Archeological Museum of Chios. Published with permission. **(B)** The raspatory (left), the serrated trepan or trephine (center), and the trepan (right) were used for scraping, sawing, and drilling, respectively. Used with permission from Tsermoulas et al. (2014). Panel **(C)** is adapted from Hanson (1999).

History is the study of the present traces of the past. If objects no longer exist, it is as though they had never existed. The essential element for historical study is the remaining evidence of materials from the past; those that no longer exist, cannot be studied (Elton, 1967). History involves analyzing and interpreting the present traces of the past and integrating information from different sources to develop a “historical memory” of events as they occurred. An example of this is the study of trepanation in ancient Greco-Roman medicine. To understand trepanation, Tullo (2010) examined the osteoarcheological evidence, the material culture and the written texts from the Roman Empire. The osteoarcheological evidence consisted of skulls, such as the skull of Chios, which showed evidence of trepanation (Figure 1). The material culture consisted of the tools used for trepanation, which were sophisticated surgical instruments for boring holes in the skull. The written texts described the procedures for trepanation. For this, Tullo (2010) consulted Hippocrates, *On Head Wounds*, written in 400 BC (Panourias et al., 2005). Each of these present traces of the past, however, had to be interpreted in the present as no physician now exists from ancient Rome to describe the process of trepanation, why and how it was done, how patients were treated and how many survived the treatment. Many other historical artifacts no longer survive. Bandages, ligatures, steel tools and other objects deteriorate with time and provide no present traces. Thus, the study of history involves the quest for the present traces of the past.

Archeological evidence and material culture are kept in museums and ancient manuscripts are kept in libraries and archives. These are the sites that select, classify and maintain the present traces of the past; the basic materials that comprise “archival memory” (Steedman, 1980; Jimerson, 2003; Rivera-Orraca, 2009). Like individual and collective memory, archives, museums and libraries are selective in what they collect and maintain; many objects are never saved, while others are lost, deteriorate or are discarded. Thus, “archival memory” is selective and subject to errors, omissions, and interpretations. Museum displays and archival documents reflect the perspectives of those who decide what to save (remember) and what to discard (forget), and how to classify, maintain and display their holdings (memories; Jimerson, 2003). Like individual and collective memory, archival and historical memories are reconstructions of the past from the present traces, as organized by historians at a particular time (defined as “the present”) and in a particular place. However, many historical artifacts are lost or destroyed. For the history of neuroscience, these losses are serious. If history is a collective memory of the past, the loss of the historical objects and documents of neuroscience is equivalent to “scientific Alzheimer’s disease.” Treating this disciplinary ailment is more than a matter of preserving and cataloging laboratory notes, specimens, equipment, drawings and documents. This material needs to be incorporated into the teaching and practice of neuroscience today.

History is not static but is updated by new discoveries. As with neuroscience itself, new discoveries enable us to update our knowledge of the history of neuroscience. New discoveries of lost manuscripts, letters or memoirs; new biographies and autobiographies of scientists and the discovery of new experimental phenomena allow us to reconsider the course of history. For example, new discoveries resulted in the re-evaluation of the function of Golgi cells and Golgi’s theories of cellular connections (Kruger and Otis, 2007; Galliano et al., 2010). Likewise, modern discoveries in epigenetics have led to the up-dating of Darwin’s theory of evolution (Skinner, 2015) and our understanding of environmental influences on brain and behavior development and evolution (Keverne, 2014; Keverne et al., 2015). Likewise, the discovery of letters from Sigmund Freud to his school friend, Eduard Silberstein, not published until 1990 (Boehlich, 1990) give new insight into Freud as a student.

### Saving the Vanishing History of Neuroscience

The present traces of the past are being lost. These include books, diaries, published articles, unpublished manuscripts, photographs, original data, laboratory books, equipment and instruction manuals on how to use this equipment. As the people, both scientists and technicians, who did the work retire and then die, the experimental protocols, technical skills, expertise and oral history is lost with them. This matters because the crucial element for studying history is the present evidence of the past; with no evidence, there is no history. To understand the history of neuroscience, we must save the traces of neuroscience of the past and present. But how should this be done? Books should be donated to and saved by libraries. Articles, manuscripts, diaries, lab notebooks and data should be sent to archives. Equipment and the instruction manuals for its use should be sent to museums. Why not develop a “History of Neuroscience Museum?” Lorusso et al. (2018) have proposed a “neuroscience without borders” program to preserve the history of neuroscience in Europe. IBRO, FENS and the Society for Neuroscience all have sections on their websites on the history of neuroscience and history poster sessions at their annual meetings. Autobiographical accounts of neuroscientists provide important information on how the history of neuroscience was done by those who did it (Squire, 1996–2018). More neuroscientists should be encouraged to write their memoirs and send their lab notes and other research materials to libraries, archives and museums.

### The Virtual History of Neuroscience?

The internet is an important resource for saving the history of neuroscience. Many historical neuroscience books and journals are now available online as are some archives. FENS, IBRO and the SfN have history websites, but what else can be done? Why not develop a virtual museum of neuroscience? This was our thinking when we developed the Oxford History of Medicine website, which focuses on the history of neuroscience^1^. On this website, we have digitized the slides from the collections of Sir Charles Sherrington and Sir Wilfrid Le Gros Clark. A variety of historical instruments, teaching models and other objects have been captured using 3-D photography and can be viewed on this website. There is also a collection of clinical neurological cases with case histories, stories about the objects, art works, and videos of Oxford lectures on the history of neuroscience.

We have also developed the European Brain Museum Tour project (Lorusso et al., 2018), which is a virtual tour of museums with specialized collections on the brain and the history of neuroscience in each country of Europe^2^. Using this virtual tour, neuroscience enthusiasts can find exhibits of interest and use the links on this website to view each museum’s own website. This project, which is now sponsored by the FENS History of Neuroscience Committee, started as part of the “History of Neuroscience” course that I taught at Dalhousie University and we hope that it will be useful to researchers as well as students in neuroscience. There are numerous other websites about the history of neuroscience, such as the milestones in neuroscience research^3^. These can be found using an internet search.

## Why Study the History of Neuroscience?

Neuroscience is a recent area of science, emerging as an explicit discipline in the late 20th century (Shepherd, 2010), but the history of neuroscience goes back to the ancient Egyptians (Breasted, 1930; Finger, 1994). Most students enter neuroscience to perform cutting-edge research with innovative technologies, to understand the complex workings of the brain and discover how to repair them when mechanisms fail. Seldom do students enter neuroscience in order to study its history. As with other scientific disciplines, neuroscientists focus on new discoveries and contemporary theories. However, even the disproved and rejected theories of the past, such as phrenology, which have been relegated to cautionary anecdotes and the introductory sections of textbooks, may be re-evaluated in light of more recent discoveries (Jerison, 1977; Jones et al., 2018).

Many neuroscientists are interested in the history of their discipline, but not everyone agrees that the knowledge of the history of neuroscience is helpful or important for doing modern neuroscience research (Falk and Falk, 2007). Neuroscientists tend to work pragmatically; using models that work and are consistent with the data without worrying about the philosophical or historical issues involved. However, theoretical models break down, and at some point, they cease to be able to explain observed phenomena or to produce new testable theories (Maienschein et al., 2008). At this point, a history of science perspective can be useful to analyze a theory historically, investigate the circumstances under which it was created, learn about its creators, examine the phenomena and the original data incorporated into it, critique the underlying assumptions, and examine rival theories that were previously rejected (Maienschein et al., 2008). This approach offers a wealth of material to research scientists, who can look to these historical findings to examine how theories are constructed. With a history of science approach, the end of a model’s productive life does not mean that it cannot still furnish important lessons to researchers. For example, there are numerous theories of the causes of Alzheimer’s disease (Armstrong, 2013; Cubinkova et al., 2018; Table 1), and new research attempts to integrate behavioral, genetic and environmental risk factors into an over-arching theory of AD (Nehls, 2016). Once the causes of AD are known, one should be able to follow the historical threads of each of these theories.

**Table 1:** What causes Alzheimer’s disease?

The amyloid cascade hypothesis (APP, PSEN 1, PSEN2)
The tau hypothesis (Tau)
The APOE4 allele hypothesis
The protein folding hypothesis
The alpha-synuclein hypothesis
The prion-like hypothesis
The synaptic pathology hypothesis
The abnormal neurotransmitter activity hypothesis (cholinergic, glutamatergic)
The neurotrophic hypothesis (BDNF)
The neuro-vascular hypothesis.
The impaired insulin, IGF-I signaling hypothesis
The cholesterol hypothesis
The neuro-inflammation hypothesis (astrocytes, microglia)
The autoimmune hypothesis
The slow acting infection hypothesis (HSV)
The mitochondrial cascade theory (rate of mitochondrial decline)
The oxidative stress hypothesis
The cell cycle hypothesis
The altered blood-brain barrier hypothesis
The trace metal hypothesis (copper, zinc, aluminum)

*There are many hypotheses about the causes of Alzheimer’s disease, but no definitive support for any of them. How will history treat the study for the causes of AD? (from: Armstrong, 2013; Cubinkova et al., 2018; Makin, 2018)*.

A history of science approach can also be an aid to understanding the interdisciplinary nature of neuroscience. Neuroscience, or more accurately, the neurosciences, involve anatomy, physiology, pharmacology, neurology, psychiatry, molecular biology, biochemistry, physics, cell biology, developmental biology, evolution, physics, chemistry, engineering, computer science, ethology, psychology and economics (Shepherd, 2010). Each of these fields has its own theories, methodologies, and data, but through collaborative research projects, these influence each other. For example, during the time that Star (1983) observed researchers in a psychophysiology laboratory, she saw that they collaborated with neurologists and imported neurological procedures into their research. In doing so, the psychophysiologists adopted the assumptions about the localization of brain function made by the neurologists. Although this interdisciplinarity is both necessary and fruitful in neuroscience (Shepherd, 2010), it comes with the risks of mixing disciplinary assumptions about neural processes without a strong grounding in the bases for these assumptions. A critical historical approach can help neuroscientists engaged in interdisciplinary research to navigate these issues (Falk and Falk, 2007).

### The Importance of the History of Science in General

To understand the importance of studying the history of neuroscience, it is useful to review the importance of historical research in science in general. To appreciate the role of science and its influence on society involves understanding its history (Nutton, 2004; Lindberg, 2007), philosophy (Godfrey-Smith, 2003) and sociology (Merton, 1962; Bloor et al., 1996). The history of science describes the discoveries in science, their evolution and how they were transmitted and received (Grainger, 1956). A textbook of neuroscience, for example, documents the history and progress of each area of neuroscience (Finger, 1994). Discoveries do not appear out of the blue; they have long periods of incubation and their importance may not be recognized for many years. New scientific knowledge is transmitted through books and journal articles, which have their own history (Gross et al., 2002). Only when they are published can new discoveries be distributed and critically evaluated.

Many new discoveries have been rejected as “nonsense” when first published only to be confirmed later (Gross, 2009), resulting in paradigm shifts in science (Kuhn, 1962). The discovery of a new fact is exciting, but if it is too revolutionary it will be rejected by established scientists; it must be proved to be correct and doubters must be convinced by hard data. The discovery of a truth is one thing, but equally necessary is its transmission to other scientists and their acceptance of it (Grainger, 1956, p. 80). New discoveries and new theories result in controversies. Of such controversies are scientific histories made. For example, Vesalius’s (1543) book on human anatomy was rejected when first published and stimulated heated controversy that lasted decades (Montagu, 1955). The debate on whether neural communication was electrical or chemical (the soups vs. the sparks debate) also continued for decades (Valenstein, 2002). The theory of animal electricity incited debates between Galvani and Volta (Piccolino, 1998) and the neuron doctrine resulted in debates between Golgi and Cajal (Guillery, 2005). The history of neuroscience is important for investigating how advances in neuroscience occur. For example, while some would argue that new tools and techniques are fundamental for new discoveries in neuroscience (Bickle, 2016), others would argue that conceptual change is what drives advances in neuroscience (Parker, 2018).

The formal academic study of the history of science dates from the founding of the journal ISIS by George Sarton in 1913 (Sarton, 1918; Barnes, 1920; Hellman, 1958) and showed a significant increase in the 1950s (Albrecht-Carrie, 1951; Grainger, 1956). By the 1970s, Brush (1974) made the suggestion that the history of science might be subversive and should be X-rated. He suggested, with tongue-in-cheek, that the study of the history of science might pervert young minds by telling them the truth about how science is done as opposed to the idealized methods of science presented in textbooks. By this time, the traditional objective view of the history of science as presented by Elton (1967) was giving way to the more subjective interpretational view of history given by Carr (1961) and represented by the view of history as a flawed memory process (Cubitt, 2007). It is interesting to compare Grainger’s (1956) reasons for studying the history of science (Table 2), which focus on discovering objective truths, to the more subjective reasons of Maienschein (2000). On the other hand, although Maienschein (2000) states that even if the history of science is subjective and riddled with errors of memory, there are still many good reasons for studying it (Table 2).

**Table 2:** Why study the history of science?

Grainger (1956).
1. To understand scientific achievements in relation to society and culture.
2. To indicate the directions and progress of science itself.
3. To illustrate the creative imagination involved in basic science.
4. To view the needs and problems of science in relation to society and education.
5. To show the long periods of innovation and development underlying new discoveries.
6. To show the struggles of new discoveries to be accepted by other scientists and society.
7. To show that what is considered “true” in science is continually being revised in light of new discoveries.
8. To describe the changes in the way that new scientific discoveries change our beliefs about the world.
9. To show how scientific discoveries underlie advances in engineering, technology and medicine.
10. To humanize science, integrating the history of science with the humanities.
11. To put current discoveries into historical perspective.
12. To evaluate the benefits and shortcomings of each side of a scientific controversy, correcting errors and pointing out contradictions equally.
13. To illuminate how a discovery in one area of science relies on knowledge from other areas.
14. To illuminate the sense, purpose and reasoning of science for education.
15. To focus a critical eye on new discoveries, evaluating them in light of past research.
Maienschein (2000).
1. To show students and the general public how science works and how to improve it.
2. To show the greatness and the weakness, the fallibility and the humanity of scientists; reassuring students that scientists are, after all, only human.
3. To show the excitement of science.
4. To illuminate why some science ‘works’ better than other science.
5. To show that science is not a static method unchanging over time, but incorporates new innovations and responds to changing environments.
6. To increase the public understanding of science and promote scientific literacy.
7. To demonstrate past failures as well as past successes in order to avoid the former and build on the latter.
8. To make us better scientists; stressing creativity and humanness. To keep scientists from being too arrogant about successes and too despondent about failures
9. To reveal the mistakes of the past and make us more efficient; recognizing mistakes prevents us from making the same mistakes again.
10. To provide the larger perspective and allow scientists to make better judgements of their own work and that of others.
11. To stimulate the imagination. Many new ideas and inventions are simply adaptations, modifications or new uses for old ideas or inventions.
12. To show how science is really done by real people who are fallible and make mistakes as well as clever discoveries, and how many discoveries are the result of errors and good luck in addition to careful observation.
13. To increase the public understanding of science. It makes science more accessible and interesting, showing the excitement of science and promotes scientific literacy.

Grainger (1956) painted an idealized view of the historian of science as someone who must have a sound knowledge of science, philosophical wisdom and a historical sense, and be able to embrace the whole picture of the role of science in society. According to Grainger (1956), the historian of science must also be aware that the education of science students focuses on teaching “facts” and technological expertise. Many scientists are so specialized and narrow in their outlook that they reject the history of science as “irrelevant” and a waste of time. The enlightened scientist, however, judges modern findings in the light of history in order to understand the relationships between seemingly disparate studies and to use knowledge from one area of science to facilitate discoveries in another. Finally, the history of science, like any history, is an exercise in detective work. In some ways understanding the history of a scientific discovery is almost as exciting as making the discovery itself (Goodfield, 1981).

Falk and Falk (2007, p. 44) suggest that there are a number of benefits of scientists becoming historians. These include the analysis of the social and cultural background of scientific discoveries; the context of scientific research; and the implicit assumptions that underlie the “dominant scientific Zeitgeist” under which scientific research is conducted. They argue that scientists “adopt unawares those doctrines (and implicit assumptions) that are established by the dominant scientific Zeitgeist,” and suggest that studying the history of science provides a way that scientists can recognize and overcome their culturally-determined biases about their research. In the past, historians of science emphasized that scientists had an almost moral duty to learn the history of science so that they could situate their pursuit of knowledge in relation to the social, cultural and political events of their time (Grainger, 1956). This is still an important reason to study the history of science. In many cases, scientists investigating the brain are unaware of the cultural biases that influence their work (Cooter, 2014). Historians of science should make, and neuroscientists should read, engaging, critical, rigorous histories of the study of the brain and the nervous system to overcome these biases. Another benefit of studying the history of science is to credit researchers whose discoveries were premature and thus unheeded, or published in older or foreign-language (non-English) journals (Gross, 2009).

Scientists who become historians also bridge the “two cultures” of the arts and social sciences and scientific research and are more able to see the influence of the arts on the sciences, and vice-versa (Edwards, 2010; Frazzetto, 2011; Garcia-Lopez, 2012). Scientists who take a historical perspective are able to have a more comprehensive perception of their scientific work and may see things that others miss (Root-Bernstein, 1988). Finally, because there is a great amount of “implicit knowledge” in science that cannot be learned from textbooks, but only from hands-on research experience, the scientist-cum-historian can explain the implicit rules of scientific work which are never included in published articles. Indeed, one of the most significant contributions of scientists to the history of science may be their personal reminiscences, stories and anecdotes about how their research was really done. These only come to light in autobiographical works such as those of Watson (1968) on the double helix and Cajal (1937/1989) on his life in neurobiology. The personal accounts of many neuroscientists (e.g., Hebb, 1980) have been given in the many volumes of *The history of psychology in autobiography* that began as far back as the 1930s and in the series of *The history of neuroscience in autobiography* edited by Larry Squire since 1996.

Finally, scientific theories influence broader societal ideas about the way the world works. The rise of the neurosciences has produced a “neuroculture,” a distinct sociocultural phenomenon that looks to neuroscience to explain all aspects of human behavior (Rolls, 2012; Mora, 2015). Neuroculture has taken hold in literature, film, television and the visual arts, as well as education and even economics (Frazzetto and Anker, 2009). Indeed, neuroculture seems to have taken hold of all aspects of modern life, but it is not without its critics (Casper, 2014; Cooter, 2014).

In summary, the answer to the question, “why study the history of neuroscience?” falls into five categories: (1) self-improvement, illuminating the theories and methods of neuroscience and improving upon them; (2) efficiency, avoiding past mistakes and learning from them; (3) perspective, providing judgment and clarity and thus enlarging the scope of neuroscience; (4) imagination, offering a wide repertoire of ideas; and (5) education, improving scientific literacy and the public understanding of neuroscience (Maienschein et al., 2008). Shepherd (2010) points out that studying the history of neuroscience helps us to understand the interdisciplinary nature of neuroscience, and shows how neuroscientific research extends across all species, systems and levels of neural organization. A historical approach examines the factors that produce discoveries: the methods and techniques used, the personalities of the scientist themselves and the social, cultural, political and ethical issues underlying neuroscience research.

## How Does History Affect You?

The average neuroscientist or student of neuroscience may not feel that they are affected by the history of neuroscience. However, the history of neuroscience is not just an academic discipline; history is personal and your own research history is important in three ways. The terminology you use, the scientific articles you write, and the integrity of your research publications all depend on history.

### Naming the Brain

Simply talking about the brain is an exercise in history. The term “brain” was first used by the ancient Egyptians in about 1700 BC (Breasted, 1930); the distinction between the “cerebrum” (enkephalon) and “cerebellum” (parenkephalis) was first made by Aristotle around 300 BC, and the “entorhinal area” was defined by Brodmann in 1909 (see Swanson, 2015 for the history of neuroanatomical terminology). Likewise, the terms “neuron,” “synapse,” and “neurotransmitter” all have a history (López-Muñoz and Alamo, 2009). Histological techniques, such as the Golgi stain have a history (Shepherd et al., 2011), the “neurone doctrine” (Shepherd, 1991/2015), the “amyloid cascade hypothesis” of Alzheimer’s disease (Hardy, 2017) and the theory of the developmental origins of health and disease (DOHaD; Hoffman et al., 2017; Suzuki, 2018) all have a history. So to talk about any aspect of neuroscience is to talk about history.

### Writing a Scientific Paper

The completion of your research articles relies on the history of the problem of interest, and, when your research article is published, it becomes a part of history. There are many guides on how to write research articles (for example: Neill, 2007; Plaxco, 2010; Adams, 2011; Kallestinova, 2011; Saper, 2015). Each section of a research article involves some form of history, thus all scientists study history when completing a research project and writing a scientific article. The “Introduction” summarizes the history of the problem that you are investigating and introduces the hypotheses you plan to test. It summarizes the main discoveries in the area and who made them. When you are testing a theory, the “Introduction” describes who developed the theory, what previous data supports or refutes it, and describes any controversies, which you hope that your research will resolve. It provides the scientific context for your research problem with reference to theoretical and empirical developments in the field. Your “Methods” section describes the apparatus and procedures used, often discussing who developed the equipment and techniques, their reliability and validity. Your experimental methods, both those explicitly referenced in the article and those tacitly passed on, all have a history. In some cases, older methods may be used to solve research problems in the present (Schwerdtfeger, 2018; Schwerdtfeger and Tobet, 2018). The core of your research article is the presentation of new results. Once the experiment is completed, your “Results” section is written and adds new data to the field of study. These results are new today, but as soon as they are published, they are history. The “Discussion” section describes how your findings fit into the history of the problem and whether or not they support the theory being tested. The “Discussion” section summarizes how your research is relevant to previous research and summarizes the advances that you have made. The “Reference” section describes the history of the problem by listing the publications that you relied on to complete your study. It is a bibliographic resumé of the history of your research topic. When writing a scientific article, you become a selective historian. Your introduction does not cite every article on your topic; it is selective, focusing on the articles most relevant to your study. Likewise, you select the apparatus, methods and procedure from the many options available. You may even select which results to publish and which to omit. Your “Discussion” is also selective, focusing on one or more of the possible theories to explain your results. Finally, your reference list is also selective; you do not cite every single article on your topic.

Once published, your article can become part of the history of someone else’s research project. If you write a review article, it is a history project. Your grant proposals are also history projects: they must demonstrate how the proposed research fits into the history of the field. Grant reviewers and journal referees must evaluate whether your research adds enough to the great chain of knowledge to be funded or published. This means that the reviewers of grants and manuscripts must know the past and current history of the research being evaluated. Thus, everything that you do in neuroscience involves history. Often without realizing it, neuroscientists study history simply by conducting their research, writing review articles, grant applications and reviewing manuscripts.

### Keeping Your Own Historical Archives

A scientific publication involves two types of history: the history of the problem being researched (the literature cited) and the history of the experimental project itself. If someone wants to replicate your experiment, they will need to know all of the details of the methods and procedure: the subjects, the protocols followed, equipment used, laboratory conditions and methodological issues not included in the article and they may want to see your laboratory notebooks, apparatus and methods, raw data, statistical analyses, etc. (Gorgolewski and Poldrack, 2016; Gilmore et al., 2017; Zwaan et al., 2018 and the ensuing commentaries). But what happens if other researchers cannot replicate your findings? What if you are accused of fraud or research misconduct? Your raw data, statistical analyses, letters, notebooks, diaries and e-mails will be open to scrutiny. Thus, for each published article, a personal archive is critical in order to provide a comprehensive documentation of your experimental procedures and results and to establish that there was no scientific misconduct, fudging of results, manipulation of data or photo-shopping of figures. Open and transparent research relies on a careful historical account of each research project (Iqbal et al., 2016). To improve reproducibility of published findings, many journals, such as Nature, require authors to submit research report forms that provide information on the research subjects, materials used, experimental design and statistical analyses (see Nature Publishing Group, 2017).

What do you do when others cannot replicate your results? This happened to me when other authors (Birke and Sadler, 1984) could not replicate the results of my study on olfaction and scent marking in rats (Brown, 1978). By carefully examining the differences in methods between the two studies, I was able to show that using the methods of my study, I replicated my results and using the methods of Birke and Sadler (1984), I replicated their results (Brown, 1991). Thus, by replicating the different methods used in both studies, I was able to show that the differences in social and sexual experience of the rats in each study produced the different behavioral results. There are many such methodological issues in behavioral neuroscience (Schellinck et al., 2010). Thus, it is important to keep a research archive for each publication.

When developing a personal research archive, many questions must be answered. What should be kept? Where should it be kept and for how long? How can other scientists access your archive? Your research archive is a historical document and may become subject to inspection and scrutiny. Many journals, universities, and governmental institutions now provide archives for the documentation of each research project and the storage of raw data. For example, Cambridge University has a website with a guide to research data management^4^, and there are a number of other guidelines to how to manage research data (see for example, Ingram, 2016).

The development of data archives enables the creation of large neuroscience databases for data sharing (Cheung et al., 2009; Freeman, 2015; Wiener et al., 2016). Researchers must, therefore, ensure that the data archived in these large scale databases are reliable and valid. Once such databases are created, those who wish to use them must learn new techniques for data-mining and neuroinformatics (Grisham et al., 2010, 2016; Akil et al., 2011; Gregory et al., 2018) and for statistical analysis (Bzdok and Yeo, 2017). Formal data archives have been developed in structural biology (Kleywegt et al., 2018) and neuroimaging (Gorgolewski et al., 2016; Borghi and Van Gulick, 2018). The Jackson Laboratory Mouse Phenome Database^5^ is a collaborative archive where data collected on laboratory mouse strains and populations have been collected from multiple sources. We used such public databases (The Allen Brain Atlas, STRING, GoMiner and Mouse Genome Informatics databases) for an in-silico analysis of grooming behavior (Roth et al., 2013). New resources have been published for making large databases available to neuroscientists (Vogelstein et al., 2018) and for searching these databases for designing and planning research projects (Matiasz et al., 2018). Thus, if your data is included in a public database, you must ensure that it is replicable.

What happens if your results cannot be replicated and you are accused of scientific fraud (Gunsalus and Robinson, 2018)? Other researchers, university administrators and granting agencies may want to inspect your personal archives for each experiment. If scientific misconduct is found (if your experimental data does not support the published results) your articles may be retracted and your reputation as a scientist tarnished. The Retraction Watch website^6^ keeps track of retracted articles. If you cannot replicate your own experiments, the published articles may also need to be retracted. For example, LoLordo and Ross (1990) retracted their own articles because the rescoring of videotapes on which the published data were based did not confirm the original findings. Zhang et al. (2012) had their article in the “Neurobiology of Aging” retracted at the request of the Editor-in-chief and the authors “due to inappropriate duplication of photomicrographs and errors in the description of the material” with the result that “the quantitative results reported in the article cannot be considered reliable” (Zhang et al., 2016). If you are falsely accused of scientific misconduct, you will need to use your scientific archive to demonstrate your research integrity (see Goldenring, 2010). On the other hand, some authors have argued that most published research is not replicable (Ioannidis, 2005), while others argue that failure to replicate is an inherent property of scientific research (Redish et al., 2018). Ioannidis (2014) has presented 12 steps for improving the reliability and validity of published research.

Even if you are certain that none of your own research articles will be retracted, you need to beware of referring to retracted articles in your research articles, reviews and grant proposals. For example, in one of our articles (O’Leary et al., 2018), we referred to Zhang et al. (2012), but when we were doing the revisions to our article, we found that this article had been retracted (Zhang et al., 2016), and so we had to find a new reference and make substantial revisions to our article. When writing review articles one must also beware of retracted articles. Uher and Weaver (2014a) wrote a commentary on a article by Perroud et al. (2014) that was subsequently retracted because one of the authors had fabricated data (Aubry et al., 2014). As a result, Uher and Weaver (2014b) had to retract their commentary on this article. In this case, the embarrassment of having to retract an article had nothing to do with the reviewers, who were innocent victims of a third party. Thus, the Retraction Watch website and database (Brainard, 2018) is becoming an essential resource for preventing neuroscientists from becoming victims of scientific malpractice by others.

In summary, history is a personal issue in neuroscience. Each of your research projects relies on previous studies, methodology and theories. Once your article is published, it becomes a part of history. Establishing a personal archive for each research project is critical to ensure that future researchers can gain access to your data and that your research documents are available for historical analysis. Such an archive is also essential for other researchers who wish to replicate your experiments and to defend yourself against claims of scientific misconduct and the danger of having your articles retracted. Finally, you must be wary of citing articles that have been retracted.

## How to Make the History of Neuroscience Relevant Today

History is more than a matter of cataloging and preserving laboratory notes, specimens, equipment, drawings and photographs. This material needs to be incorporated into the teaching and practice of neuroscience research. To make history relevant to students of neuroscience, faculty could integrate historical topics into their neuroscience teaching and research using websites, artifacts and historical publications. Through the study of history, students can be introduced to the historical context of their research and learn how it is integrated with other disciplines and society in general. The history of neuroscience can be approached through textbooks (Finger, 1994, 2000; Glickstein, 2014), or books on special topics (Shepherd, 1991/2015). There are biographies of many neuroscienctists, including Galen (Mattern, 2013), Helmholtz (Meulders, 2010), Golgi (Mazzarello, 2010), and Pavlov (Todes, 2014). Neurobiographies (Söderqvist, 2002, 2007) and autobiographies (Squire, 1996–2018) provide personal histories, describing how individual neuroscientists did their research, and bridging the gap between neuroscience and the humanities.

The most common way that neuroscientists do historical research is to identify past studies on the topic of interest using reference citations, PUBMED, Web of Science or Google Scholar as a guide. However, this often does not encompass the many texts located on library shelves or in library special collections of rare books that must be accessed separately from the main library catalog. Archives keep letters, diaries, photographs, memos and other documents donated by scientists or their families. They may include drafts of articles, unpublished manuscripts, lab data books, and other valuable information. Newspaper clippings, awards, medals, and honorary degrees also find their way into archives. For example, the McGill University archives have many boxes of articles of Donald O. Hebb (Brown and Milner, 2003) and the Oxford University archives have the articles of Sir Charles Sherrington, but so do the archives at the University of Liverpool, the University of British Columbia and the Royal Society of London (Molnár and Brown, 2010). The National Archives of each country also contain materials on the history of individual neuroscientists.

Historical objects may be kept in museums, and there are many museums with brain collections that provide essential resources for studying comparative neurobiology and the history of neuroscience (Iwaniuk, 2010, 2011; Fobbs and Johnson, 2011; Manger, 2011 and other articles in this volume). The importance of saving anatomical specimens in museum collections is that they can be re-analyzed using modern methods to gain new insights into neural disorders. Such is the case of the brains of Broca’s famous patients, Leborgne and Lelong, held in the Museum Dupytren in Paris, which have been reanalyzed by modern researchers using PET and fMRI scans to reveal the damage that was unseen by Broca (Dronkers et al., 2007). In addition, the old equipment used in scientific laboratories, including machinery and lab notes, become critical historical objects (Ceccarelli, 2002; Arnold and Söderqvist, 2011) and it is important to preserve these in museum collections (Lorusso et al., 2018).

Beyond these physical locations, information on the history of the neurosciences is readily accessible through the internet. The internet is an incredibly powerful tool for the study of the history of neuroscience and for engaging students in historical research. Even the most ancient texts can be accessed online. There are also virtual archives and virtual museums. The European Brain Museum Tour website^7^ allows anyone to locate museums with brain collections at the click of a button. Developing a virtual museum of neuroscience was the reasoning behind the creation of the Oxford History of Medical Sciences Project^8^ where students and researchers can access slides, objects, art, stories, case histories and seminars that are relevant to conducting historical research. There is also the ability to analyze historical equipment using this website.

Historical research also involves personal interactions: contacting the colleagues, students, friends and families of neuroscientists to locate hidden information about their lives and work. Former students and colleagues of neuroscientists may have letters, manuscripts and photographs stored away in filing cabinets. There may be old materials in university store-rooms that have never been sent to the archives. Still, other historical artifacts have been stored in back rooms of university departments, or in display cases in corridors. Other documents reside in the basements and attics of the friends and families of neuroscientists. Interviews with colleagues, students, spouses, children and grandchildren of neuroscientists may turn up unseen documents, as well as valuable information that has never been published. For example, in this way I obtained some letters and other documents from the families of Sir Wilfred Le Gros Clark and Donald Hebb. Such family documents are not publically available and are on the verge of being lost if someone is not there to salvage them. Once all such information has been collected, one must sort and arrange it and decide how to preserve it. Too often there are large gaps, missing information and one-sided conversations. One may find the letters from A to B, but none of the letters from B to A, and so inferences must be made about the information they might contain. Additionally, if the topic that you are researching is controversial, you may find yourself the center of a war of words, as I found with my investigation of Donald Hebb’s research on sensory deprivation (Brown, 2007a).

History is the study of the present traces of the past or the memories of the past. Thus libraries, archives, and museums are all repositories of the memories of the history of neuroscience. However, we are now facing a number of problems in preserving the history of neuroscience (Lorusso et al., 2018). Many of these repositories have selective memories; some select only certain items to keep and others have political and personal preferences for what is collected. Some archives have online indexes and others do not. Some archives no longer have archivists. Some archives and museums have been closed and the materials are no longer accessible. In addition, libraries, archives and museums “get rid of” or de-access items they no longer deem interesting. Old books, just the kind of thing a historian looks for, which have not been taken out of the library for years, get discarded, so are no longer available to anyone. Archival materials sit in boxes without being indexed and museums reject donations of old equipment as they have no space for it. Most museums exhibit only a small fraction of their holdings; the rest is in storage and may become lost. And, worst of all, documents and equipment may have been destroyed or thrown out by over-zealous administrators who just want to “get rid of all of this old junk.” Finally, many neuroscientists do not leave their records to any library, archive or museum and these records get destroyed or languish unknown in attics and basements. My aim in this article is to encourage the preservation of these historical documents.

## The Excitement of Historical Research

Historical research is interesting, illuminating and enlightening. To be able to conceptualize the perspectives of older neuroscientists gives us an ability to understand their discoveries more deeply and make sense of our own research, theories, and methodology. In addition, historical research allows one to hear exciting stories and meet interesting people. If these are not appealing as reasons to study history, some may find consolation in the fact that doing historical research often involves travel to incredible places. Some examples from my own research illustrate how the search for historical documents can be interesting and exciting: academic detective work. These include finding ancient Greco-Roman surgical tools in Greece, searching for Leonardo da Vinci in Italy, finding Sir Charles Sherrington’s box of slides in Oxford, the search for traces of Donald O. Hebb in Montreal, Boston, and Chicago and the opportunity to visit Pavlov’s laboratories and home in Russia.

### Ancient Greco-Roman Surgical Tools

**Figure 2:**
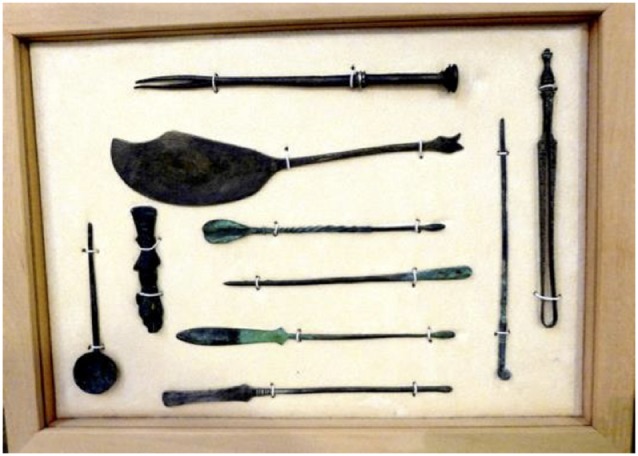
Surgical tools from the 3rd century AD. These tools are replicas of those on exhibit at the National Archelogical Museum of Greece and were purchased at the museum in Thessaloniki, Greece. On the far left is a copper-alloy spoon for preparing and taking medicines and applying them to wounds. Next to it is a copper-alloy knife handle that is decorated with a small animal, possibly a mouse, which links the instrument with Asklepios. The mouse was seen as a daemonic being with prophetic powers and was associated with Apollo Smintheus, who protected people against evil and epidemics. The blade is missing. Because blades were made of iron, they often rusted away. The item at the top of the six items shown horizontally is a copper-alloy double hook which is decorated with silver bands at the head and in the middle. It was used during surgical operations on blood vessels (aneurysms), on membranes in the eye, and on tonsils, and to clasp pieces of tissue and the edges of wounds during surgery. The second horizontal item is a copper-alloy knife used to make incisions in the flesh during operations. It has an engraved depiction of a snake on the blade and a snake’s head at the end. The third item is a copper-alloy spoon-shaped probe that was used to prepare and apply medicines. The fourth is a copper-alloy needle used to sew bandages applied to wounds. The fifth is a copper-alloy spatula probe used to mix and apply medicines in deep surgical incisions, to diagnose and measure the depth of injuries, and more rarely to clean internal wounds to the nose and other, larger wounds. The bottom item is also a copper-alloy spatula probe. On the far right is a copper-alloy clasp with serrated ends. This was used to clasp or cut away flesh and tumors during surgical operations. Next to this is another copper-alloy spatula used to clean wounds and incisions, scrape away fistulae, and remove foreign bodies and broken bones from the ear and nose. In eye operations, it was used to remove cysts. It was also used to prepare and apply medicines, particularly to the eyes. These tools are described by Bliquez (1982).

During a FENS meeting in Thessaloniki, Greece, in October 2015, I visited the Archeological Museum, which had exhibits on trepanning and on ancient surgical tools. Sets of exact replicas of these tools were for sale in the gift shop and I bought one to use for teaching (Figure 2). These tools include hooks, knives, probes, needles, spatulas, spoons, and knife handles. A copper-alloy knife, used to make incisions during operations, has an engraved depiction of a snake on the blade and a snake’s head at the end, which link the instrument to Asklepios, the god of medicine. A scalpel handle carved in the shape of a mouse is also linked to Asklepios (see Figure 2). When I lecture on the writings of Hippocrates or Galen, I open the box of ancient surgical instruments and the students are struck by how “modern” they look. This increases their interest in history.

### The Search for Leonardo

**Figure 3:**
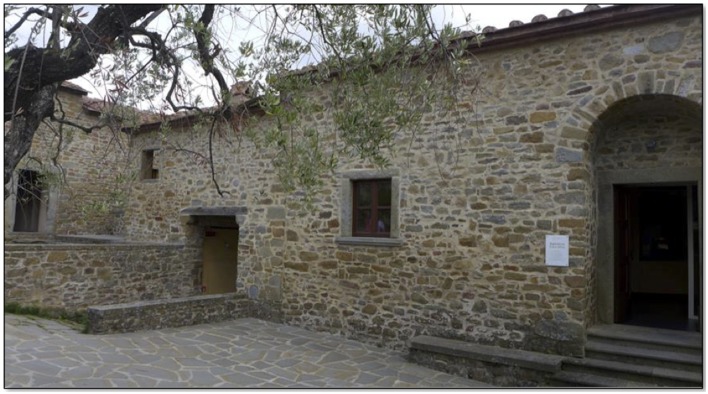
The house in Vinci, Italy, where Leonardo Da Vinci was born [Photo by Richard Brown].

In order to complete a presentation on Leonardo da Vinci for a history poster (Brown, 2014), I obtained security clearance to view the original pages from Leonardo’s notebooks, which are held in the print room at Windsor Castle in England. When I left I ordered my own copies of the prints to use for teaching. During the FENS Milan meeting, Lorenzo Lorusso organized tours of libraries and archives such as the Biblioteca Nazionale Braidense, which displayed Leonardo’s drawings. On this tour, we visited the town of Vinci (Figure 3), the Leonardo museum and the house where Leonardo was born. In Florence, we visited other Leonardo da Vinci museums, libraries, and exhibits where we purchased books on Leonardo and prints of his drawings. In researching Leonardo da Vinci as a neuroscientist, a tour of Italy is interesting and exciting.

### Sherrington’s Box of Wonders

**Figure 4:**
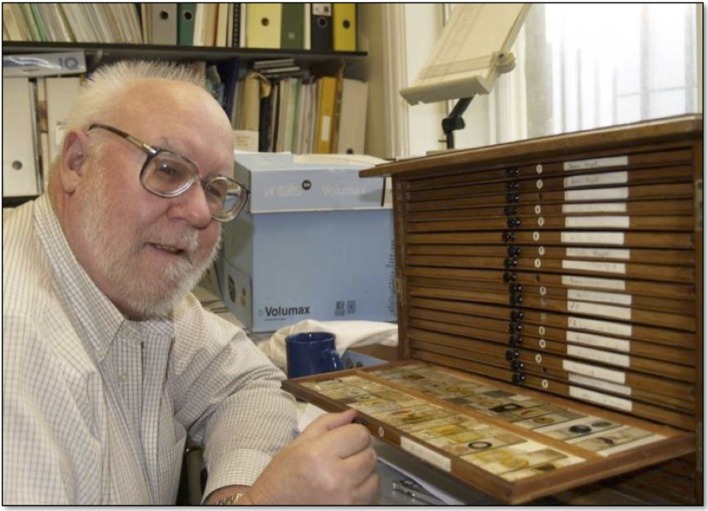
I am examining Sherrington’s box of slides at Oxford University [Photo by Zoltán Molnár].

In 2008, Zoltán Molnár discovered a box of histological slides used by Sherrington from 1888 to 1935 (Figure 4). This box contains 21 drawers of slides from Sherrington’s years at St. Thomas Hospital (1888–1895), Liverpool University (1896–1914) and Oxford University (1914–1935). It also contains slides presented to him by other leading contemporary neuroscientists. Much of the histological data behind these incredible discoveries are available for research at the University of Oxford. This material provided information for our publication on the work of Sherrington (Molnár and Brown, 2010) and the impetus to develop our History of Neuroscience Website^9^. This website allows the viewer to use a program called “Zoomify” to magnify each image as if it were under a microscope. The materials in this website can now be used for teaching classes anywhere in the world (Chang and Molnár, 2015).

### The Man Behind the Hebb Synapse

**Figure 5:**
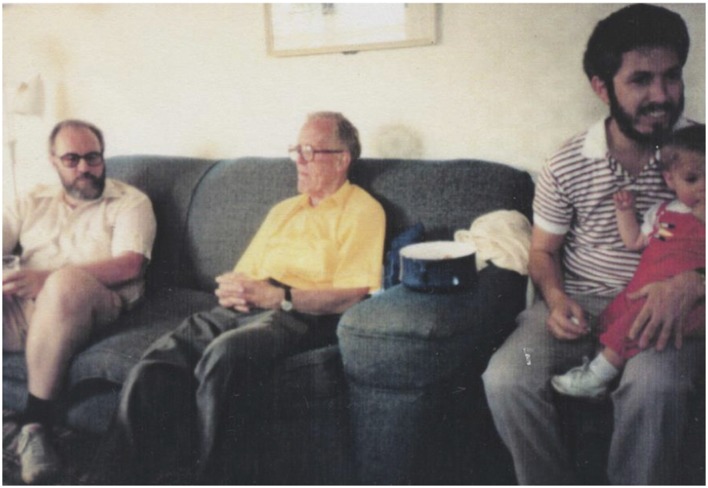
At the Hebb “cottage” in Chester Basin, Nova Scotia. Hebb’s 80th birthday 1984. Left to right: Richard Brown, Donald Hebb, Raymond Klein and his daughter [Photo taken by John Fentress].

Donald O. Hebb’s influential book, *The Organization of Behavior* (Hebb, 1949/2002), established the concepts of synaptic change and cell assemblies. This was the basis for the prominent Hebbian theory, which has been summarized as “cells that fire together wire together.” Hebb’s research was diverse and involved a wide range of ideas (Brown, 2007b) and his research on intelligence A and B was used by Cattell to develop his theories of fluid and crystallized intelligence (Brown, 2016). Hebb was Professor Emeritus at Dalhousie University from 1977 to 1985 (Figure 5) and, after he died, a Hebb Memorial Lecture was established. I began to write the introductions to these lectures and this resulted in having Hebb’s book republished (Hebb, 1949/2002) and writing articles on his life and work (Brown and Milner, 2003; Brown, 2007b). To research the life of Donald Hebb took me to the archives at Dalhousie University, McGill University, the University of Chicago, Harvard University and the archives of the History of Psychology in Akron Ohio, as well as to many small archives and museums. Hebb’s family provided me with materials and I met his brother, Andrew (aged 98) who told me about their childhood and schooling. I scoured the National Archives of Canada in Ottawa for information about Hebb and met with his former students and colleagues and I am not finished yet; indeed, I have my own “Quest for Corvo” (Symons, 1934/1966) in trying to write a biography of Donald Hebb.

### Pavlov’s Physiology Labs

In May 2017, the FENS History committee participated in the 100th anniversary of the Russian Physiological Society in Saint Petersburg Russia. During our visit, we not only presented lectures but also visited the laboratories used by Pavlov. The highlights of the trip were our visits to the lab at Koltushi and to Pavlov’s apartment, where we had tea in Pavlov’s dining room (Brown et al., 2017).

These examples illustrate some of the excitement of the search for the history of neuroscience. It is interesting because you discover things that you would never have expected. It is illuminating as it shows the ideas and methods used to make some of the basic discoveries in neuroscience. Finally, it is enlightening to view the lives of famous neuroscientists through their writings, letters and photographs and through the eyes of their students and families. In the search for the history of neuroscience, you have the opportunities to meet interesting people, hear fascinating stories and to travel to far away places. Finally, the study of the history of science helps you to make sense of your own research.

## Summary and Conclusions

The history of neuroscience can be important for students and researchers who can use insights from the history of science to illuminate their work. By methodologically investigating historical data, models, hypotheses and experiments, alternatives to contemporary theories can be contemplated. Lessons from the history of neuroscience also reveal the cultural context and social responsibility of those investigating the brain. Popular ideas about the brain influence the direction that neuroscientists take in their research. Especially in the past half-century, new discoveries in neuroscience have had a widespread popular appeal. Nerves and brain function have become a powerful analogy in spheres of thought far removed from neuroscience. In order to avoid repeating prejudices, neuroscientists can take a history of science approach to their discipline.

Because history points out the flaws and problems with past research, it has been suggested that it be “X-rated,” as the focus has moved away from the search for the “objective truths” in history to the more subjective “memory” approach to history, with all its errors, omissions and flaws. Since all research relies on history, and each research project has its own history, neuroscientists rely on their historical records to demonstrate their research integrity. Without history, whether in the form of actual physical objects, written documents, or personal reminiscences, neuroscientists have little context for their contemporary work. Historical approaches can be integrated into research and teaching in neuroscience and many neuroscientists will find interest and pleasure in the study of the history of neuroscience.

## Author Contributions

RB wrote the manuscript.

## Conflict of Interest Statement

The author declares that the research was conducted in the absence of any commercial or financial relationships that could be construed as a potential conflict of interest.

## Footnotes

^1^https://history.medsci.ox.ac.uk/

^2^https://www.fens.org/Outreach/History/EBM/

^3^https://faculty.washington.edu/chudler/hist.html

^4^https://www.data.cam.ac.uk/data-management-guide/organising-your-data

^5^https://phenome.jax.org

^6^http://retractionwatch.com

^7^https://www.fens.org/Outreach/History/EBM/

^8^http://history.medsci.ox.ac.uk

^9^https://history.medsci.ox.ac.uk/
